# Spatial clustering in vaccination hesitancy: The role of social influence and social selection

**DOI:** 10.1371/journal.pcbi.1010437

**Published:** 2022-10-13

**Authors:** Lucila G. Alvarez-Zuzek, Casey M. Zipfel, Shweta Bansal

**Affiliations:** Department of Biology, Georgetown University, Washington, District of Columbia, United States of America; The Rockefeller Foundation, UNITED STATES

## Abstract

The phenomenon of vaccine hesitancy behavior has gained ground over the last three decades, jeopardizing the maintenance of herd immunity. This behavior tends to cluster spatially, creating pockets of unprotected sub-populations that can be hotspots for outbreak emergence. What remains less understood are the social mechanisms that can give rise to spatial clustering in vaccination behavior, particularly at the landscape scale. We focus on the presence of spatial clustering, and aim to mechanistically understand how different social processes can give rise to this phenomenon. In particular, we propose two hypotheses to explain the presence of spatial clustering: (i) *social selection*, in which vaccine-hesitant individuals share socio-demographic traits, and clustering of these traits generates spatial clustering in vaccine hesitancy; and (ii) *social influence*, in which hesitant behavior is contagious and spreads through neighboring societies, leading to hesitant clusters. Adopting a theoretical spatial network approach, we explore the role of these two processes in generating patterns of spatial clustering in vaccination behaviors under a range of spatial structures. We find that both processes are independently capable of generating spatial clustering, and the more spatially structured the social dynamics in a society are, the higher spatial clustering in vaccine-hesitant behavior it realizes. Together, we demonstrate that these processes result in unique spatial configurations of hesitant clusters, and we validate our models of both processes with fine-grain empirical data on vaccine hesitancy, social determinants, and social connectivity in the US. Finally, we propose, and evaluate the effectiveness of two novel intervention strategies to diminish hesitant behavior. Our generative modeling approach informed by unique empirical data provides insights on the role of complex social processes in driving spatial heterogeneity in vaccine hesitancy.

## Author summary

In recent decades, vaccination hesitancy behavior has gained ground, threatening the maintenance of herd immunity. Geographic clustering of vaccine hesitancy creates pockets of unprotected sub-populations that can become hotspots for outbreak emergence. Even though the complex phenomenon of vaccine hesitancy has been well-studied by the scientific community, the mechanisms driving geographic clustering in this behavior are poorly understood. In this study, we consider two hypothesized mechanisms that may lead to geographic clusters of high hesitancy: (a) we hypothesize that clusters of high hesitancy arise because hesitancy is associated with underlying socio-economic traits (e.g. high income) that are themselves geographically clustered; or (b) we hypothesize that clusters of high hesitancy arise because hesitant individually social diffuse the behavior among their social network which tends to be geographically clustered. We use computational experiments to test the impact of these mechanisms on the geographical distribution of hesitancy behavior. We find that both processes are independently capable of generating geographical clusters of high hesitancy, and propose and evaluate the effectiveness of two novel intervention strategies to slow the rise in hesitancy and diminish the increased risk of outbreak emergence due to geographic clustering.

## Introduction

Vaccination is the single most effective way of mitigating the consequences of infectious diseases, and has resulted in steep declines in infection, morbidity, and mortality for vaccine-preventable diseases (VPDs) [1, 2]. To manage VPDs, high levels of vaccination coverage are critical to controlling outbreaks. However, vaccine uptake and the maintenance of herd immunity have been threatened in recent decades due to vaccine hesitancy behavior, which is the delay or refusal of vaccination despite its availability, and is influenced by factors such as complacency, convenience, and confidence [3]. Vaccine hesitancy behavior has increased worldwide, leading the WHO to declare it as one of the top global health issues [4]. In high-resource settings like the United States, vaccine hesitancy for childhood vaccinations has been found to be concentrated in major metropolitan areas [5] with substantial evidence linking recent VPD outbreaks of measles and pertussis in urban populations with hesitant behavior [6, 7]. More recently, vaccine hesitancy has also been jeopardizing the suppression of the COVID-19 pandemic [8–11]. Continued work is thus urgently needed to better understand the characteristics and dynamics of hesitancy behavior so we may stem the tide of increasing hesitancy and prevent the threat it poses.

A critical complexity to our ability to diminish growing hesitancy is the distribution of the behavior within a population. Vaccine hesitancy has been shown to not be randomly distributed in populations but instead occurs in clusters of individuals in the same geographic areas. This geographic heterogeneity poses a major challenge to the control and elimination of VPDs, as it leaves pockets of unprotected sub-populations that may be vulnerable to outbreaks, even if they are part of a population with high average vaccination coverage. Past research has identified spatial clustering of vaccine hesitancy and geographic overlap between hesitancy clusters and clusters of reported disease cases [12–14]. Modeling work has also demonstrated that the presence of geographic clustering in hesitancy can drive increased disease emergence risk and transmission [15–17]. Such spatial heterogeneity in hesitancy can be driven by complex social processes, but our understanding of the structure and impact of such processes remains limited.

Past meta-analytic work has proposed two broad categories of social determinants of hesitancy behavior in individuals (distinct from vaccine-specific issues such as safety or cost): contextual influences, arising from social, cultural, environmental, and geographic factors; and group influences, arising from personal perception or the context of a social or peer environment or experience [3]. Based on these determinants, two social processes have been proposed that may generate behaviors such as vaccine hesitancy: a) *social selection*: in which socio-cultural determinants independently drive hesitancy behavior. Under this hypothesis, individuals that share those social characteristics interact with each other due to shared traits and independently engage in hesitancy behavior [18–20]; and b) *social influence*: in which vaccine hesitancy behavior propagates over existing social networks. Under this hypothesis, individuals that engage in hesitancy behavior influence others among their social contacts to do the same [21, 22].

Significant work has considered social selection and social influence theoretically and empirically for vaccine hesitancy [23–25] as well as other behaviors [26–29]. However, all previous studies have focused on the individual-scale, in which individual attributes or behavior diffusion among individuals drives changes in behavior. What remains to be considered are the dynamics of social selection and social influence at the landscape-scale (i.e. across a large spatial area like an entire country made up of individual communities), in which the attributes of communities and collective behavior diffusion due to social connectivity between communities might drive dynamics of hesitancy behavior. As public health strategies are driven by a population health perspective, and because the geographic distribution of vaccination is critical to disease emergence across spatial scales, it is important to study dynamics at the landscape-scale beyond individuals [14]. At the same time, considering vaccination at a fine spatial scale is also critical as vaccine uptake can be heterogeneous within and between large geographical areas and public health policies are most commonly implemented by local jurisdictions [16, 30, 31].

In this study, we aim to characterize the role of social selection and social influence leading to spatial clustering in vaccination hesitancy at the landscape scale. While vaccine hesitancy is often considered generally, we define vaccine hesitancy in a vaccine-specific manner, and assume that hesitancy is strongly correlated with uptake for the specified vaccine [32] (which may only hold for mandated vaccines in high-resource settings). We hypothesize that landscape-level spatial clustering in hesitancy may be produced by (a) social selection: geographically proximate communities may independently adopt similar behaviors because they share characteristics that promote the behavior; or (b) social influence: vaccination behavior may diffuse between socially proximate areas through learning of social norms and practices. In the case of social influence we expect to see that clustering in vaccination behavior arises surrounding highly hesitant populations, whereas under social selection, the clustering in vaccination behavior only reflects geographic clustering in underlying drivers. Using a spatial network framework in which we consider the social and physical interactions between communities, we use a generative approach to introduce a specified level of social selection (based on community socioeconomic characteristics) or social influence (driven by the direct diffusion of behavior). To balance the importance of spatial heterogeneity on public health policy-making and implementation, we develop our model for the entire United States at a fine-grain (US county level). Based on a distribution of hesitancy behavior, we then classify communities according to vulnerability to disease emergence: communities with a high level of vaccine hesitancy, putting them below the vaccination herd immunity threshold, are considered vulnerable; otherwise, they are considered protected. For a given proportion of vulnerable nodes, we mechanistically seek to understand whether social influence or social selection can independently (and together) generate spatial clustering patterns, and to what degree. We additionally seek to understand how the structure of the network over which these social processes occur impacts the generation of spatial clustering in hesitancy. We empirically validate our models using fine-grain data on vaccine hesitancy, social determinants, and social connectivity in the US. And, based on our findings, we propose and evaluate strategies to diminish overall levels of hesitancy in communities, as well as reduce spatial clustering in hesitancy. Our work integrates theoretical models with empirical data to develop a mechanistic understanding of the dynamic processes that potentially generate geographic clustering in hesitancy behaviors and informs interventions to ameliorate this dangerous behavior.

## Materials and methods

Our work aims to characterize how social processes affect the spatial distribution of vaccine hesitancy at a landscape-scale and translate this understanding for mitigation strategies to diminish the spatial clustering present in hesitancy behavior across the US. In order to achieve this, we develop a theoretical spatial network model that allows for tunable spatial structure and social process dynamics. We parameterize this model with empirical data to validate our findings, and use a simulation approach to assess the effectiveness of proposed mitigation strategies.

### Empirical data

We integrate three empirical data sources into our work for model parameterization, validation and application, further described below: (1) we specify an infection case study, and use relevant empirical vaccine hesitancy data based on school exemptions; (2) from the US Census, we identify each county’s socioeconomic attributes relevant to our social selection model; and (3) based on social media activity data, we define social connectivity between communities relevant to our model of social influence. Our models are national and includes all US counties. All our data sources are at a fine spatial scale (US county), but they vary in their coverage (ranging from 17 US states to all US states).

#### Disease case study and vaccine hesitancy data

The principles of herd immunity suggests that if a large portion of a community is immune to disease due to vaccination, further transmission is unlikely. The proportion of the population that needs to be vaccinated to achieve herd immunity depends on how contagious the pathogen is. For our work, we focus on measles as a case study. Because measles is highly transmissible (*R*_0_ ≈ 20), the herd immunity threshold, *ρ*, for measles is *ρ* = 1 − 1/*R*_0_ = 0.95 [33]. Thus, an estimated 95% of a community needs to be vaccinated to achieve herd immunity for measles (we refer to such communities as *protected*). On the other hand, any community that cannot achieve 95% vaccination coverage due to vaccine hesitancy can expect sustained outbreaks, we refer to these communities as *vulnerable*. State-mandated school entry immunization requirements in the United States play an important role in achieving high vaccine coverage for measles, but variations in vaccine exemption policies result in a patchwork of vaccine coverage across the country. All states allow exemptions for medical reasons and the vast majority also allow parents to opt out of childhood vaccination mandates due to non-medical religious or philosophical reasons. To measure vaccine hesitancy, we thus use data on non-medical exemptions for school children (S2 Fig) [34], which is available for download at [35]. These data were collected from state health departments, and have been collated into a unified dataset at the county-yearly level for 17 states in the US. To parameterize hesitancy in our social selection and social influence models for all US counties, we use these data to estimate that vaccine hesitancy behavior at the county level follows an exponential distribution, *P*_(*η*)_ = λ *e*^−λ*η*^ (S1 Table). We assume that the data from these 17 states is representative of all US states given the political, socio-economic and geographic heterogeneity in the sample.

#### Socioeconomic data

Previous work has shown that hesitancy behavior for childhood vaccinations such as the measles-mumps-rubella (MMR) vaccine is associated with socioeconomic traits. In particular, smaller average household size and larger average household income levels have been shown to be positively correlated with increased hesitancy behavior [36, 37]. We thus use these two socioeconomic traits in our social influence and social selection mechanistic models as well as our validation and mitigation models, based on county-level data from the US Census (shown in S6 Fig) for all US states.

#### Social connectivity data

To model empirical social (rather than physical) connectivity between communities (particularly for the social influence process), we use the Facebook social connectedness dataset [38], available for all US states. A pair of communities (i.e. US counties) are connected if Facebook users in one community are “Facebook friends” with users in the other community. The social connectedness index (SCI) captures the strength of the interaction. We created SCI-thresholded networks (SCI > 400) based on a weighted network analysis (S10 Fig). At SCI > 400, we find that the average degree of the network is 6 and it contains 2496 counties.

We also find that the structure of the social connectivity is both spatial (i.e. communities are connected with their geographic neighbors) and aspatial (i.e. communities are connected with geographically distant communities). While social media connectivity does not capture all social connections between communities, the significant spatial structure of this network suggests that this connectivity may reflect interactions between communities in the physical world which tend to be geographically structured. The structure of this data also suggests that the landscape social network may be well approximated by a small-world network model, which represents networks with significant spatial connections and occasional aspatial connections. To confirm this, we measure the small-worldness coefficient *σ* ≈ 178 (≫ 1) and ensure the empirical network exhibits small-world characteristics [39, 40].

We thus use a Watts-Strogatz small-world network as the structure of our social influence and social selection mechanistic models, evaluating the entire range of small-world structures, from fully spatial (rewiring probability, *p* = 0) to fully aspatial (*p* = 1). For the mitigation model we also infer the rewiring probability of the social connectivity data. To do so, we estimate the average clustering coefficient (*c* ≃ 0.29) and the average shortest path length (*l* ≃ 3.8) of the social connectivity network at a threshold of SCI > 400, and compare it to the the network properties of a small-world network with varying rewiring probabilities. From this, we estimate a small-world rewiring probability of *p* ≈ 0.2 for the social connectivity dataset.

#### Empirical estimates for model validation

To validate our models, we need data on the evolution of vaccine hesitancy. For this, we aggregate data from 2015 and 2018 for vaccine hesitancy, socio-economic traits and social connectedness (as described above). Because the datasets have non-overlapping coverage, we arrive at data on 184 counties (nodes), belonging to a total of 4 states (Arizona, California, Maine, and Virginia). (We note that these counties are a convenience sample based on data availability, but do reflect the distribution of hesitancy nationally (S5 Fig)).

We find that hesitancy behavior has been increasing nationally over recent years: 9% ± 0.2% of counties in our dataset were vulnerable (i.e. the proportion of the population not hesitant is below the herd immunity threshold) in 2015, which increases to 25% ± 1% of counties as vulnerable in 2018. At the community level, we estimate an average increase in hesitancy per county of 1% from 2015 to 2018 (S1 Table).

### Generative landscape network model

**Fig 1:**
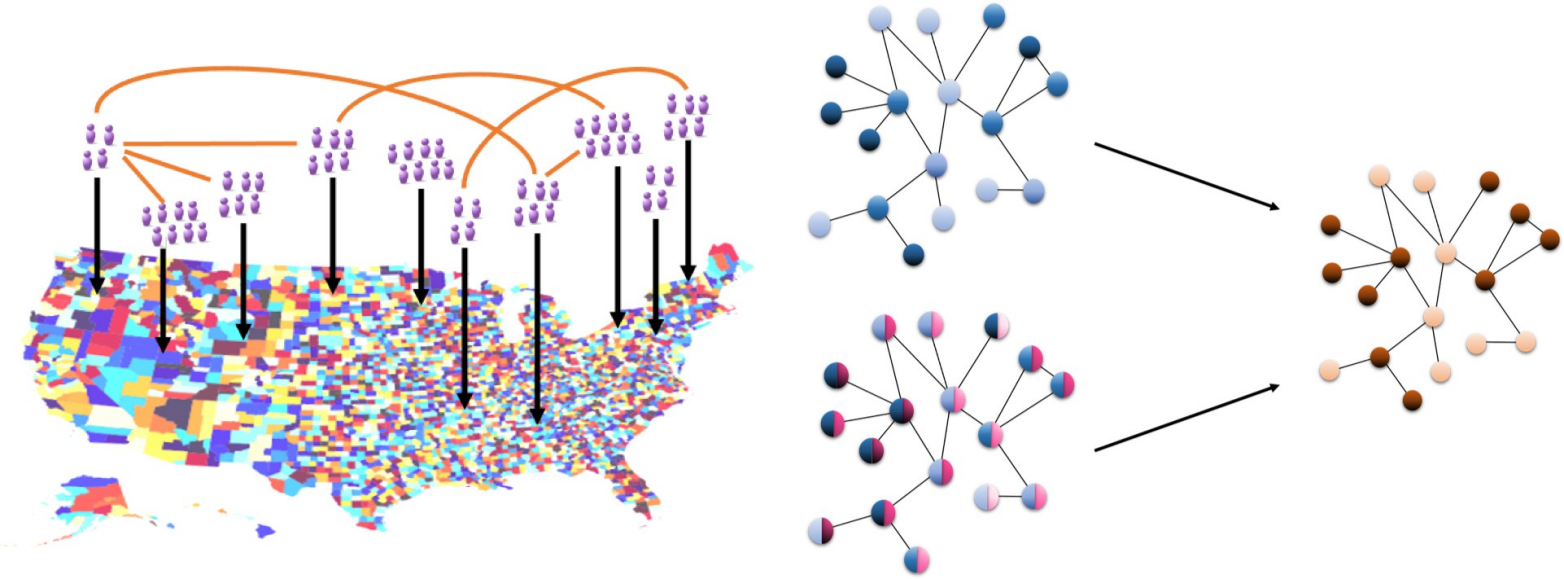
(a) A schematic of the landscape-level spatial network in the United States. Counties, which contain populations of individuals, are represented by nodes, and interactions between the county populations determine the edges of the network. (b) A schematic of the change in vulnerability due to the social influence and social selection processes. Under social influence (top arrow), each community (node) starts with a level of hesitancy (denoted in blue in the top network), and hesitancy behavior spreads through the network across social interactions (edges) between communities from hesitant (dark blue) communities to non-hesitant ones (light blue). Under social selection (bottom arrow), communities with similar traits (denoted in pink in the lower left network) tend to be connected, and some traits independently lead to hesitancy behavior (denoted in blue in the bottom left network). Each process can give rise to the same distribution of vulnerability to outbreaks (dark orange = vulnerable community, light orange = protected community in the right network).

We consider a society made up of spatially and socially interconnected communities. We represent this society as a landscape-level network in which communities (i.e. US counties) are represented by network nodes and spatial proximity or social interactions between communities are represented by network edges (Fig 1A). We define spatial proximity based on shared land borders between US counties.

We develop our landscape network model to capture structure relevant to the two social processes of interest. Social selection at the landscape-level describes the tendency of communities with similar attributes to be geographically proximal and connected. As individual and community attributes have been demonstrated to be associated with vaccine hesitancy behavior, social selection leads to spatial clustering in hesitancy due to spatial clustering in attributes. Conversely, social influence at the landscape-level describes a diffusion of hesitancy behavior over social connections between communities. These social links can be spatial (due to physical mobility, e.g. commuting behavior) or aspatial (due to social interactions, e.g. online social media) in nature. Such network structure is theoretically well-represented by the Watts-Strogatz small-world network model [41], which allows for tunable spatial network structure while preserving other network structure features such as average connectivity. For our theoretical characterization of the role of social selection or social influence in generating spatial clustering, we generate an ensemble of Watts-Strogatz small world networks of size *N* = 2048, average degree *k* = 6 (informed by the analysis of the empirical social connectivity data), and spatial structure varying from spatial (*p* = 0) to aspatial (*p* = 1) to evaluate a range of network structures.

For each social process, we use a Monte Carlo simulations to generate varying levels of the process on our spatial network model. Then, we consider how the distribution of hesitancy due to social selection versus social influence leads to spatial clustering, or pockets of high hesitancy communities in geographic proximity. For social selection, we keep the proportion of vulnerable nodes constant, but shift the configuration of vulnerability, driven by homophily in attributes. For social influence, hesitancy behavior is propagated between communities, directly increasing the proportion of vulnerable communities. In order for the two processes to be comparable, we restrict the proportion of vulnerable communities that can be generated through social influence to match the proportion assumed for social selection.

To measure spatial clustering, we calculate the level of spatial assortative mixing [42] in the community herd immunity status (vulnerable/protected) where the network is the landscape-level spatial network (i.e. edges between communities due to spatial proximity). Hence, we use

$$\begin{array}{c} r=\frac{\left(VV+PP\right)-[{\left(VV+VP\right)}^{2}+{\left(PP+VP\right)}^{2}]}{1-[{\left(VV+VP\right)}^{2}+{\left(PP+VP\right)}^{2}]} \end{array}$$ (1)

where *VV* measures the fraction of all spatial proximity edges that are between vulnerable nodes, *PP* is the fraction of all spatial edges that are between protected nodes, and *VP* measures the fraction of all spatial edges that are between vulnerable and protected nodes.

Thus, high levels of spatial clustering indicate a strong tendency of vulnerable nodes to be connected creating pools of susceptible individuals.

### Modeling social selection at the landscape scale

We model social selection in a landscape-scale spatial network in which communities are connected to each other due to geographic proximity, and community attributes are modeled based on a level of social selection. In particular, each network node (community) is described by a set of attributes associated with hesitancy behavior (representing, for example, average household income and average household size).

We begin by initializing each node with a level of vaccine hesitancy, based on an exponential distribution (*η* ∼ Exp(λ)) based on the observed data. We parameterize λ using the observed distribution of hesitancy in 2018 (with approximately 25% of all communities as vulnerable). The vector of attributes for each node is then defined by ***X*** = *η*^1/*γ*^. This makes the traits Weibull distributed with the following parameterization: ***X*** ∼ Weibull(***γ***, λ^−1^).

We then measure changes in the attribute distribution across nodes using the Mahalanobis distance, *μ*, which is defined as a dissimilarity measure between two random vectors of the same distribution with a covariance matrix ***S***. For a network with a set of edges, *E*, and attributes ***X***_*i*_ for node *i*, we defined the global distance in the network as:

$$\begin{array}{c} \mu =\frac{\sum_{(i,j)\in E}\mu_{i,j}}{\left|E\right|}=\frac{\sum_{(i,j)\in E}\sqrt{{\left(X_{i}-X_{j}\right)}^{T}\;S^{-1}\;(\left(X_{i}-X_{j}\right)}}{\left|E\right|} \end{array}$$

Based on this distance, we define the *social selection* parameter, *β*, as the deviation in distance in the simulated scenario (*μ*) from the distance in a random scenario ($\tilde{\mu }$) in which attributes are distributed randomly and independently, without correlation: $\beta =1-\left(\mu \right)/\left(\tilde{\mu }\right)$.

Our model aims to reach a desired level of social selection, β* and achieves this through an attribute randomization algorithm. The attribute vectors of nodes are swapped, and if the swap succeeds in increasing *β*, it is retained. Attribute randomization continues until *β* = β*.

### Modeling social influence at the landscape scale

We propose a social influence model that simulates diffusion of hesitancy behavior via social contacts between communities. Our model is inspired by bootstrap percolation [43], and it proceeds as follows: Each community (network node) is initialized with a level of hesitancy *η* sampled from an exponential distribution. Nodes are classified based on the herd immunity threshold (*ρ*) as vulnerable (*η* ≥ (1 − *ρ*)) or protected (*η* < (1 − *ρ*)). For our simulations, we choose measles as a case study and assume *ρ* = 0.95. At each time step, each node *i* has its own level of hesitancy and is exposed to the hesitancy levels of its neighbors, *j*. If the average hesitancy level for the node and its neighbors (avg({*η*_*i*_, *η*_*j*_∀*j*})) exceeds a tolerance (*α*), then the node’s hesitancy increases by *δ* (which we parameterize as 0.01 based on the observed hesitancy data). We refer to *α* as the *social influence parameter*, and define it as the minimum exposure required to increase a community’s hesitancy. For any protected nodes, if their new hesitancy level exceeds 1 − *ρ*, then they become vulnerable.

Our model is initialized with 10% of all communities being vulnerable and hesitancy propagation is permitted until 25% of all communities are vulnerable (parameterized based on our observed hesitancy data). This restriction is added to make the social influence process results comparable to those from the social selection process.

### Model validation

For model validation, we estimate parameters for social selection and social influence, and simulate the social influence process on the empirical dataset. We estimate the empirical social selection parameter (β*) based on the empirical distribution of socio-economic traits (average household size and average household income) and the definition of the parameter as specified below. We estimate the empirical social influence parameter (α*) as the average hesitancy level of the social contacts (as defined by the social connectivity data) of nodes which were observed to increase in hesitancy from 2015 to 2018. For both parameters, we produce uncertainty estimates using 1000 bootstrap networks of 70% of the original network size (184 counties). We also calculate the empirical spatial clustering as defined below using 1000 bootstrap samples of the original sample size (184 counties).

### Mitigation strategies

Understanding the role of social selection and influence in the spatial clustering in vaccine hesitancy allows us to develop effective intervention strategies to reduce both hesitancy and spatial clustering in hesitancy. We design strategies where specific communities can be targeted to reduce hesitancy levels. We evaluate this strategy on a theoretical social network with spatial structure parameterized based on the Facebook social connectedness dataset as a Watts-Strogatz small-world network with rewiring probability *p* = 0.2 (as described in the Empirical data section). We measure the effectiveness of each strategy in terms of a reduction in spatial clustering compared to the control case of no action.

#### Reducing clustering through social selection

For our first strategy, we propose to reduce clustering caused by social selection. Because social selection relies on a significant association between community attributes and hesitancy, we seek to target the distribution of attributes themselves. In particular, our strategy aims to select a potential vulnerable county given its level of attributes (which is positively correlated with hesitancy) and the conformity in those attributes among neighboring communities. Thus, for each county *i* we define an index, *ζ*_*i*_, as,

$$\begin{array}{cc} \zeta_{i} & =Hesitancy\;Triggers\times \;Comformity\\ & =Exp[\sum_{u\in \text{traits}}Z\left(X_{u}^{i}\right)]\times [\sum_{j\in \text{neigh}(i)}\mu_{i,j}/k_{i}{]}^{-1} \end{array}$$

where $Z(X_{u}^{i})$ is the z-score of the attribute *X*_*u*_ for the focal county *i*, and *μ*_*i*,*j*_ is the Mahalanobis distance between the attributes *i* and each neighbor *j*. This measures the conformity of the neighborhood of *i*. To implement the strategy, we target nodes with the 10% highest *ζ* values, by changing vulnerable status to protected. In the absence of hesitancy data, the primary purpose of this strategy is to efficiently detect vulnerable counties to invest resources to break hesitant clusters.

#### Reducing clustering through social influence

For our second strategy, we focus on our results which have demonstrated that small changes in network structure have a substantial impact on spatial clustering in hesitancy caused by social influence. Thus for this strategy, we aim to reduce clustering caused by social influence by altering the network structure: specifically, we target and reroute edges (i.e. social interactions over which social influence occurs) between vulnerable and protected communities with probability *ω* (constant across all edges). The rerouting strategy is carried out within the social influence model, and occurs whenever a node changes status to becoming vulnerable (due the ongoing influence process). Any edges that the vulnerable node has with protected nodes are disconnected from the vulnerable node and rerouted to a randomly selected protected node (so a vulnerable-protected edge is replaced with a protected-protected edge). In this way, further propagation of hesitancy from the vulnerable node to protected nodes can be minimized.

## Results

### Spatial clustering can be generated by social selection or social influence

Social selection and social influence have been demonstrated to be possible mechanisms driving vaccination hesitancy among individuals. Here, we examine theoretically how these two social processes can generate spatial clustering at the landscape scale. To achieve this, we consider Watts-Strogatz small-world networks with tunable spatial structure, and develop two mechanistic models to generate a tunable level of social selection or social influence in these networks. With this generative approach, we seek to characterize spatial clustering under the two social processes and in light of different degrees of spatial structure.

**Fig 2:**
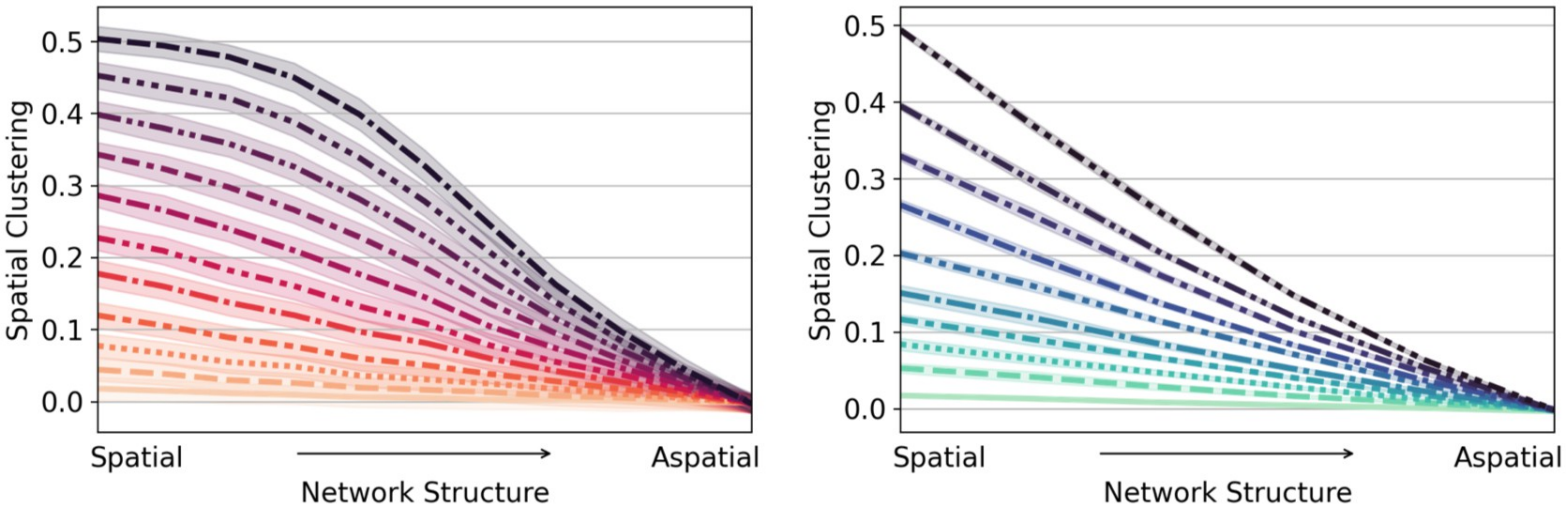
Spatial clustering generated by social selection (left) and social influence (right). For each process we show the spatial clustering in vaccine hesitancy as a function of the network structures, from highly spatial (ring) to aspatial (random) networks. Each curve corresponds to a different intensity of the given social process, from high (top, darker shades) to low (bottom, lighter shades).

In Fig 2, we illustrate that the spatial clustering can be generated by either social influence or social selection. For both processes, low values of influence/selection result in little spatial clustering in vaccination hesitancy; but as the intensity of either process increases, spatial clustering also increases. Additionally, in both cases, increased spatial structure tends to favor spatial clustering. Small changes in the structure of community connectivity lead to proportional changes in clustering with social influence, while moderate to high levels of spatial structure do not drastically change spatial clustering with social selection.

We also analyzed how the spatial nature of networks size S6 Fig. Specifically, we evaluated how the spatial structure in the network impacts the reach of a possible disease outbreak, due to the spatial clustering of hesitant behavior. Even though both social processes have different mechanisms generating their spatial clustering, we found that the maximum possible outbreak size is similar, although there is much more variation for the case of social influence.

### Social selection increases spatial clustering only in affectable societies

Social selection and social influence are driven by different social mechanisms and are expected to co-occur in complex societies. Thus, we next develop a model to consider the presence of social selection and social influence simultaneously to generate spatially clustered hesitancy behavior. Our model starts with a desired level of social selection, and allows hesitancy behavior to spread using our social influence model. With this model, we explore two types of societies: i) an affectable society is one with a low social influence parameter (*α*), in which individuals are receptive to anti-vaccine messaging and quickly adopt hesitant behavior, and ii) a determined society with a high social influence parameter, in which individuals are resistant to anti-vaccine messaging and are less likely to adopt hesitant behavior. In all cases, we allow the influence process to continue until a maximum (25%) of communities are vulnerable, as we are interested in understanding the spatial configuration of the same proportion of vulnerable communities under different scenarios.

**Fig 3:**
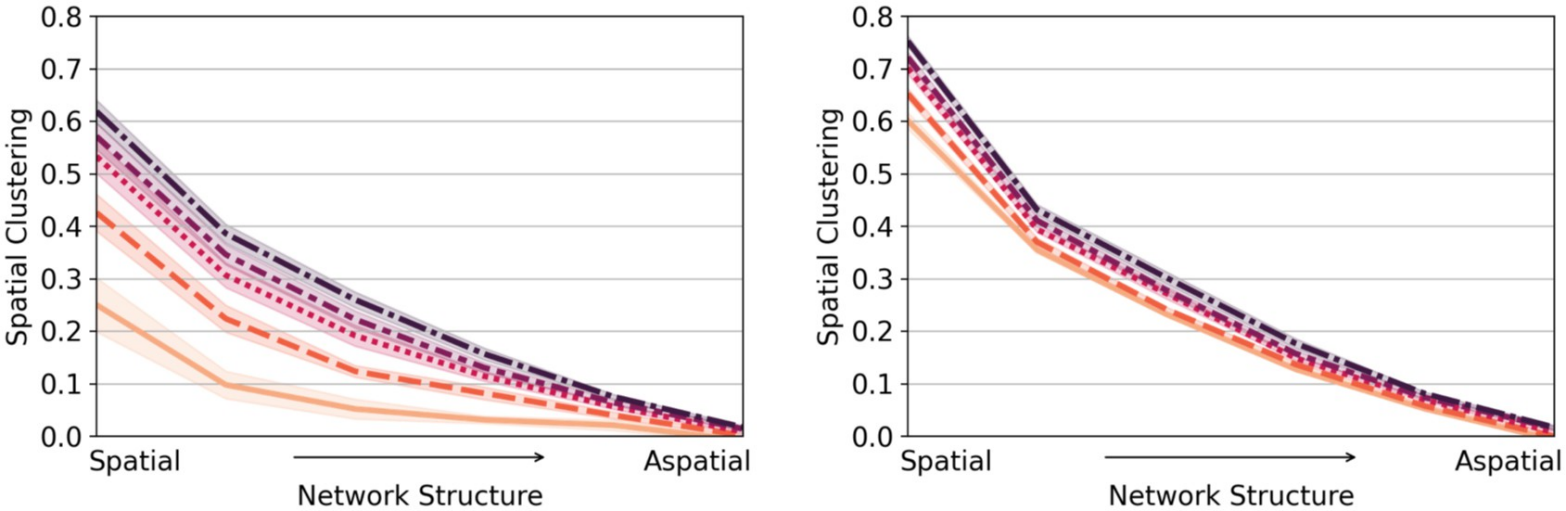
(a) An **affectable society** with *α* = 0.2, which has a low social influence parameter and (b) A **determined society** with *α* = 0.6 which as a high social influence parameter. Each curve corresponds to a different level of initial social selection; from homophilous (dark) to random (light) networks.

In Fig 3, we show the level of spatial clustering generated by social selection combined with social influence in the affectable versus determined societies. In an affectable society (low social influence parameter *α*), individuals tend to adopt hesitancy behavior easily, and we find that social selection has a strong impact in determining spatial clustering, particularly for more spatially structured societies. For a society with high social selection, as clusters of high hesitancy already exist, social influence favors their increase. For low values of social selection in an affectable society, there are few clusters, and as the population tends to turn hesitant easily (low social influence parameter), numerous small clusters are rapidly created throughout the network. In a determined society (high social influence parameter *α*), on the other hand, communities are more resistant to adopt a behavior against vaccines: to become hesitant, high consensus is needed for hesitancy among a community’s neighbors. Therefore, only few larger cluster sizes prevail.

Furthermore, we find that in the affectable society, social selection plays an essential role in the dynamics, while in the determined society, the influence process is so strong that the initial configuration of the system is not significant. We can conclude that affectable societies are more prone to create a larger number of smaller cluster sizes. In contrast, skeptical societies result in a small number of larger cluster sizes and efficiently spread hesitancy.

### Our generative model captures observed patterns of hesitancy

We evaluate and validate our approach with observed vaccine hesitancy data based on childhood school exemption data from the states of California, Arizona, Maine, and Virginia during the years 2015–2018 [34].

To consider social selection, we characterize the empirical spatial distribution of the socio-economic traits of average income and household size shown to be associated with hesitancy behavior. To consider social influence, we use empirical data on social connectivity between US counties and the empirical distribution of hesitancy for childhood vaccination.

**Fig 4:**
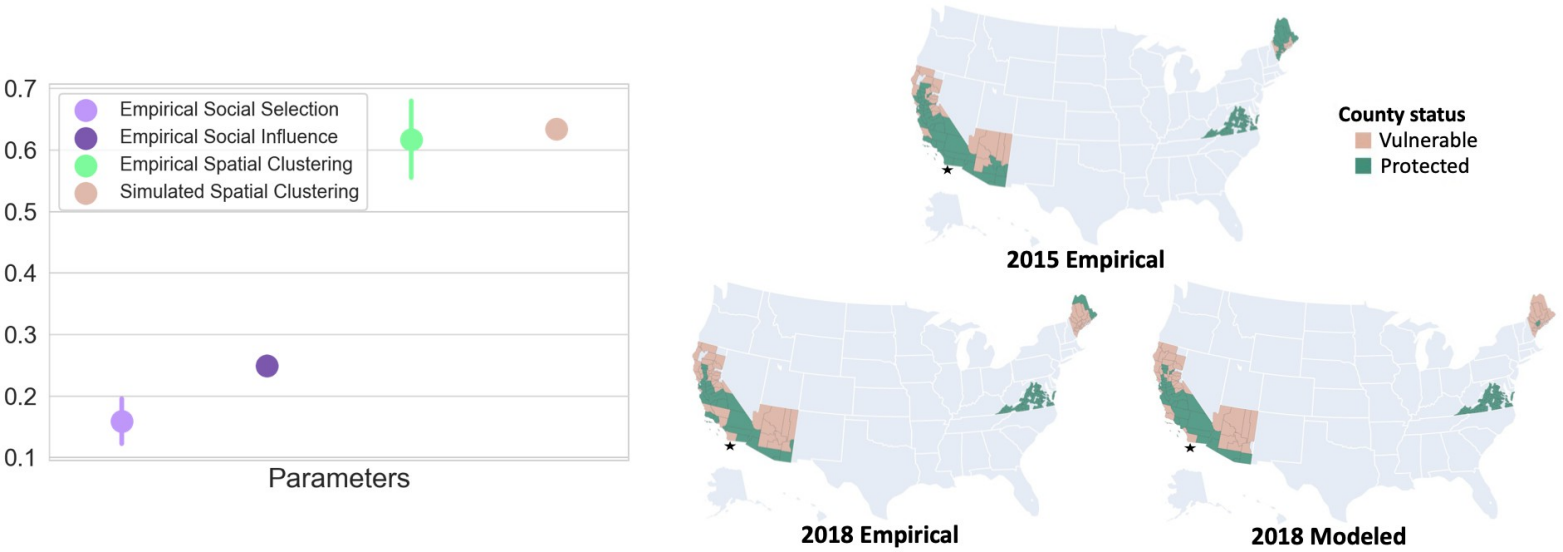
(a) We show estimated social selection *β*, social influence *α*, the spatial clustering values based on observed hesitancy data, and that from stochastic simulations of our theoretical model. b) Pink colored counties (●) are vulnerable (high hesitancy) and green colored counties (●) are protected (low hesitancy). We illustrate the observed data for 2015, alongside the observed and social influence modeled estimates for 2018. The star marks the county of San Diego, California immediately to its north.

In Fig 4A, we estimate the social selection parameter *β* based on the geographic distribution of socioeconomic attributes to be β* = 0.165. We also estimate the social influence parameter as α* = 0.253. In S9 Fig, we highlight that empirically, influence is not homogeneous geographically but displays significant heterogeneity due to spatial clustering. We estimate the empirical spatial clustering for 2018 as 0.61, suggesting that the observed spatial distribution of hesitancy is heterogeneous.

To evaluate our social influence model, we consider the spatial extent and distribution of vulnerability as observed empirically and as estimated by our model. In Fig 4B we display the observed patterns of vulnerability in both 2015 and 2018. We compare these with the estimates of vulnerability from our social influence model based on a social influence parameter of *α* = 0.253. Compared statistically, the empirical and modeled (based on an average of 1000 model estimates) spatial distributions have an F-score of 0.925. We also estimate the modeled spatial clustering as 0.625, making it consistent with the observed spatial clustering. This comparison suggests that social influence is indeed capable of generating spatial configurations of vulnerability similar to observed spatial data, based on a complex social network which has both spatial and aspatial links. To highlight this point, San Diego county in California (highlighted in Fig 4B is shown to be not vulnerable nor spatially connected to vulnerable communities in 2015. However, in 2018, it becomes vulnerable potentially due to social interactions with communities not in its geographic vicinity.

### Spatial clustering in hesitancy can be diminished through interventions

Informed by an understanding of the impact of each social process on generating spatial clustering in hesitancy theoretically and empirically, we now propose and assess the effectiveness of intervention policies. The considered policies not only reduce the prevalence of hesitancy, but also reduce spatial clustering in hesitancy, with the goal of reducing pockets of vulnerable communities rather than simply eliminating isolated counties of high hesitancy.

Social selection is driven by two key features: (a) a strong correlation between a socioeconomic trait and hesitancy, leading to high levels of the trait driving high hesitancy; and (b) a conformity or similarity in traits among neighboring communities. Hence, we propose our first intervention strategy (‘target social selection’) to diminish spatial clustering in hesitancy behavior by targeting both high levels of hesitancy-correlated traits and conformity in those traits. Because vaccine hesitancy data is difficult to obtain and may not always be available, our proposed strategy offers an alternative without the need for intensive data collection.

To evaluate how effective our social selection strategy is, we compare it with: i) a best case scenario (‘target high hesitancy’) in which available hesitancy data is used to target communities with the highest levels of hesitancy; ii) a worst case scenario (‘target randomly’) in which communities are targeted at random.

**Fig 5:**
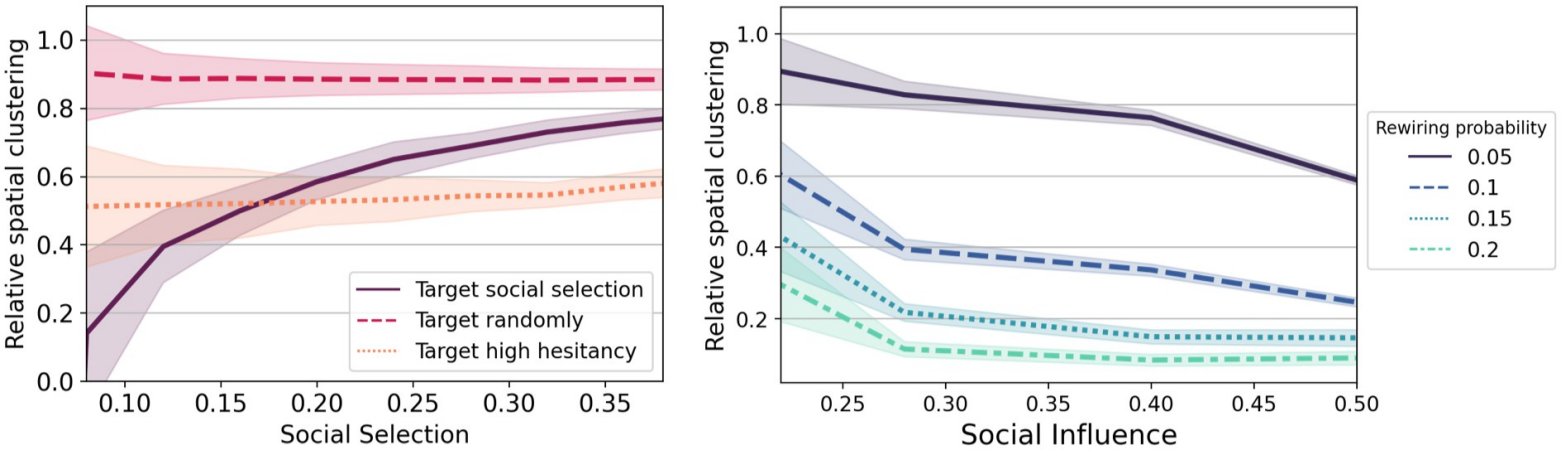
Reduction in spatial clustering via interventions, relative to no intervention: (a) We consider the impact on relative spatial clustering under the social selection strategy, which targets nodes to intervene on (target randomly, high hesitancy, high social traits) for societies with increasing levels of social selection (b) We consider the impact on relative spatial clustering under the social influence strategy, which reroutes edges with different probabilities of edge rerouting in societies of increasing levels of social influence.

In Fig 5A (and S8(A) Fig), we show that all targets under the social selection strategy are effective in diminishing clustering relative to no intervention. Because the two comparison strategies (target randomly, target high hesitancy) do not act directly on spatial clustering, their impact barely changes with increasing social selection. On the other hand, targeting social selection by targeting traits and conformity results in a larger reduction in spatial clustering for lower values of social selection. For high values of social selection, spatial clustering is higher with our ‘target social selection’ strategy than with the ‘target high hesitancy’ strategy. Within a context of high segregation, the best strategy is merely to target high hesitant counties, which implies having available hesitant data. If the level of social selection is relatively low (as we find in our empirical analysis, *β* ≈ 0.16), our findings suggest that our strategy will perform similarly to targeting high hesitant counties.

For our second strategy, we exploit the impact of network structure on spatial clustering. We seek to reduce clustering by altering the structure of the social connectivity between communities by rerouting social connections to make them less spatial. In Fig 5B, we find that as the social network is made less spatial, there is a larger reduction in spatial clustering, and that this impact increases with larger values of social influence.

## Discussion

Vaccination hesitancy is a dangerous behavior that threatens the maintenance of herd immunity, and spatial clustering of this behavior amplifies outbreak potential even when the behavior is rare. In this work, we have evaluated the role of social selection and social influence leading to spatial clustering in vaccination hesitancy. Previous literature has shown these two processes as strong candidates to be a mechanism behind this hazardous behavior. Thus far, these factors have been well studied at an individual level, and here we expand this individual-level focus to a landscape perspective, where influence and selection are processes occurring among communities within a country.

Transitioning to this large population perspective is, as data collection is feasibly and routinely done at this scale, and state-level and national public health policies are designed and implemented at this scale. To achieve this, we use a complex network approach to describe the spatial structure of counties in the United States, and we develop two generative models that describe influence and selection. Our theoretical findings suggest that both social processes generate hesitant behavior clustering, but are configured differently. In network structures ranging from spatial to aspatial, social selection tends to be a more robust process where significant changes in the network structure are needed in order to impact the spatial clustering. On the other hand, spatial clustering in hesitancy driven by social influence depends exponentially on the network structure. Thus, small changes in the distribution of edges have an impact on the spatial clustering.

Past work has demonstrated that exposure to a hesitant neighborhood in online social networks leads to hesitant behavior, so we hypothesized that both social processes are likely to co-occur. Our theoretical findings of the combined processes suggest that when the a society trusts hesitancy propaganda, social selection plays an important role and many smaller clusters of hesitancy appear. On the other hand, when a society tends to be more skeptical about propaganda, social influence overcomes and a few larger clusters appear, despite the same overall frequency of vulnerable communities. Our empirical validation conversely highlights how social influence can take advantage of a society already affected by social selection to spread hesitancy easily, generating observed patterns of spatial cluster distribution. Our case study of four states (California, Arizona, Virginia, and Maine) is a convenience sample, but may be generalizable as it reflects the distribution of hesitancy nationally (S5 Fig), includes socio-political and geographic variation, as well as diverse public health settings (e.g. California has recently made significant policy changes around childhood vaccine exemptions), and are connected via inter-state spatial and social connectivity at small and large scales. Understanding this difference in cluster configuration and distribution is critical to prediction of outbreak potential [16], and suggests the benefit of incorporating the social context of a community in public health programs.

To aid in the development of effective public health mitigation strategies, we take advantage of our theoretical results. We proposed two intervention strategies to reduce not only vaccine hesitancy levels, but to also reduce spatial clustering in hesitancy to have a disproportionate decline in outbreak potential. Our past work highlights that public health policies can affect both vaccine uptake as well as the spatial distribution of vaccination and must be implemented with caution [32]. Our strategy to reduce clustering caused by social selection focuses on targeting communities that are vulnerable due to their own socio-economic traits but also are surrounded by a socio-cultural environment with a high tendency towards hesitancy. Once the communities are identified, traditional public health measures of reducing vaccine hesitancy, such as healthcare provider training and community health outreach programs, can be implemented. Our results demonstrate that when social selection in a society is low, our strategy outperforms the strategy of directly targeting counties with known levels of high hesitancy. By simply targeting high hesitancy counties, we may be reaching isolated highly-hesitant nodes surrounded by protected communities to whom they pose little danger. Additionally, we note that even in high-resource settings such as the US, fine-grain national data on vaccine hesitancy continues to be poorly measured and inaccessible [34], thus the advantage of our social selection strategy is that it does not require access to any direct data on hesitancy. On the other hand, the success of this strategy relies on studies that identify clear associations between community traits and hesitancy behavior. Significant such work exists based on survey and interview studies of parents, social experiments, and fine-grain ecological studies, and we must continue to invest in such work to characterize the evolving socio-cultural landscape of hesitancy. Our strategy to reduce clustering caused by social influence proposes to manipulate the spatial connectivity that underlies the social influence process for communities with observed high hesitancy. While such a strategy is likely to be impractical in traditional social networks, the social connectivity between communities due to social media usage may be amenable to manipulation by expanding upon the geo-targeted and connection-targeted digital marketing techniques that are already common. Such processes are also being considered for political mobilization [44] and health applications [45, 46], and future studies could experimentally evaluate the effectiveness of such a strategy [47].

Our work has some limitations. Our social influence model design is based on findings carried out in online social networks at an individual level. Even though there is some evidence of peer influence between communities for health behaviors, we believe more evidence in vaccination hesitant sentiment is needed. Due to the lack of data, we also assume that socio-economic traits remain constant, thus the level of social selection remains constant in our models. We do note that we expect social selection to be a slower process compared to social influence, as socio-cultural environments tend to evolve slowly, thus we do not believe this to be an unrealistic assumption. We also acknowledged that the social connectivity data we use is likely not a representative sample of the total population, but expect this bias to primarily affect the edge weights of the social connectivity network (which we do not use) rather than the edges themselves. Lastly, we recognize the need for more empirical validation of our findings, and we advocate for more data to be collected on vaccine hesitancy at a fine-grain and across the United States and for a range of vaccines. The COVID-19 pandemic has shone a new light on the benefits of vaccination and the dangers of vaccine misinformation, and we hope this leads to sustained attention on these important public health areas.

We highlight that while we choose the case study of measles to ground our analysis and validation, we expect our methodology and findings to be generalizable. Spatial clustering in vaccination patterns has been found for a range of vaccines (e.g. pertussis [13], Hepatitis B [48], polio [49], human papillomavirus [50], and COVID-19 [51]). The mechanism of social selection has been demonstrated through associations of vaccination with socio-economic factors and media environment in a variety of vaccination settings [52], and evidence exists for social influence being relevant to a number of vaccines [53]. We developed our models so that they can be adapted to different vaccination systems. In the case of our social selection model, traits can be customized based on evidence for a particular vaccine, as past literature suggests that many socio-economic factors can be associated with vaccination clustering, and in fact, the same trait, such as income, can be negatively and positively associated to vaccination in different contexts [52]. Our analysis also suggests the social selection model is robust to variation in the association between traits and hesitancy and that social selection can drive spatial clustering even when the association is weaker (S10 Fig). The social influence model, on the other hand, can be adapted based on a pathogen’s herd immunity threshold and the social influence parameter, parameterized based on how pliant a society is. For a fast-evolving and complex vaccination setting like COVID-19 vaccination, for example, we might include medical distrust or political mistrust as a trait in the social selection model [54–56], and we might model the spread of misinformation-fueled vaccine hesitancy with our social influence model parameterized based on a society’s exposure to misinformation [57]. (We firmly acknowledge the current variant landscape of SARS-CoV-2 makes discussions of herd immunity complex, and such a model would be a simplification.

Understanding how social behavior impacts spatial clustering is challenging, but the methods and our findings are a step forward towards understanding the underlying processes that generate these clusters, both theoretically and empirically, and designing mitigation strategies to reduce clustering of vulnerable populations. Generative models of social behavior such as ours can also inform dynamical behavior-disease models which have been limited to assuming vaccine hesitancy in a non-spatial context and only through the lens of social influence [58–61]. The threat that vaccine hesitancy poses to local elimination of vaccine-preventable childhood diseases is growing, and we advocate for continued progress on mathematical modeling of this phenomenon from both a social and spatial perspective.

## Supporting information

S1 Fig Map representation of the level of childhood vaccination hesitancy in the US in 2015. (TIF)

S2 Fig Representation of different network structures varying the probability of rewiring edges. (TIF)

S3 Fig Distribution of vaccine hesitancy levels in the US for the years 2015 and 2018. (TIF)

S4 Fig Distribution of vaccine hesitancy levels in the US compared with the same distribution for the four states in our validation analysis. (TIF)

S5 Fig Distribution of traits associated with vaccination hesitancy in the united States for the year 2014. (TIF)

S6 Fig Maximum outbreak size generated by social selection (left) and social influence (right). (TIF)

S7 Fig Theoretical results for the spatial clustering when mitigation strategies are applied. (TIF)

S8 Fig Heterogeneity in the influence among neighbors of vulnerable (active) and protected (non-active) communities. (TIF)

S9 Fig Correlation between traits and vaccine hesitancy as a function of social selection. (TIF)

S10 Fig The Facebook network analysis. (TIF)

S1 Table Increment in hesitant behavior for the period of years 2015–2018. (PDF)
