# Supplementary figures and images for: Spatial clustering in vaccination hesitancy: The role of social influence and social selection
Source: PLoS Comput Biol. 2022 Oct 13;18(10):e1010437. doi: 10.1371/journal.pcbi.1010437 (PMC9562150; doi:10.1371/journal.pcbi.1010437)

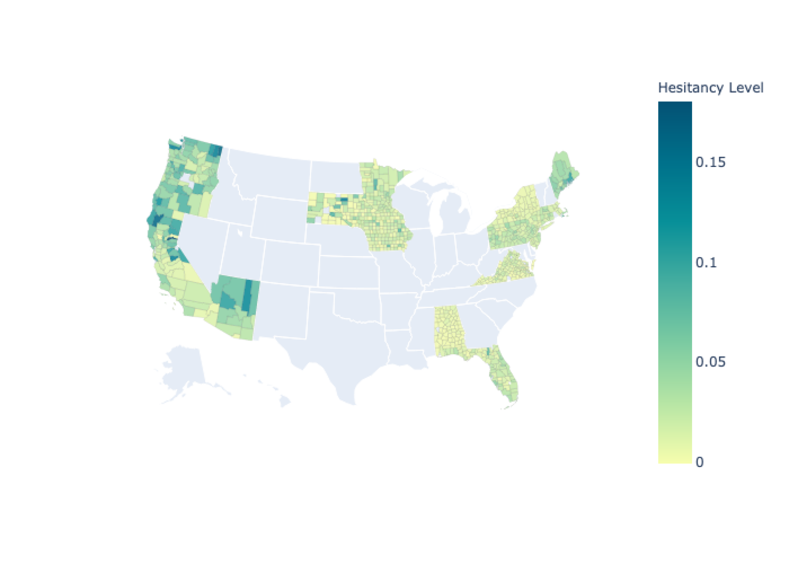

Supplement: S1 Fig — (TIF) [file pcbi.1010437.s001.tif]

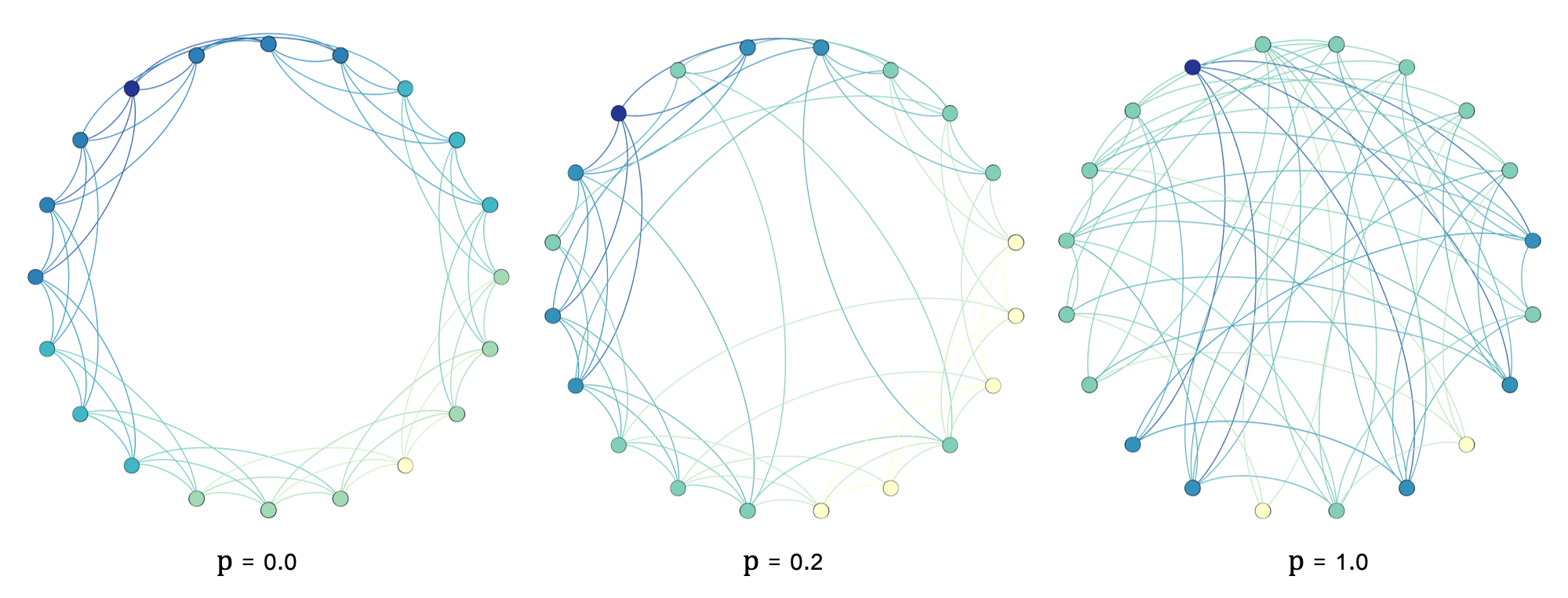

Supplement: S2 Fig — (TIF) [file pcbi.1010437.s002.tif]

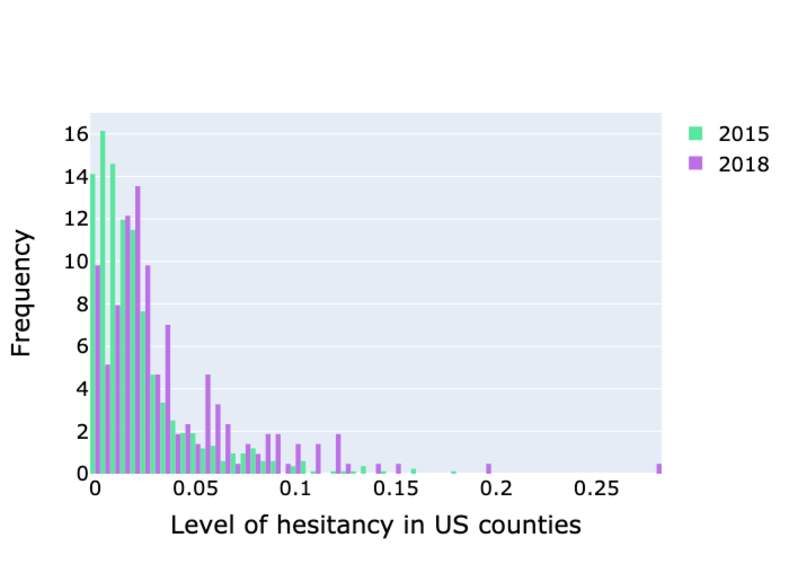

Supplement: S3 Fig — (TIF) [file pcbi.1010437.s003.tif]

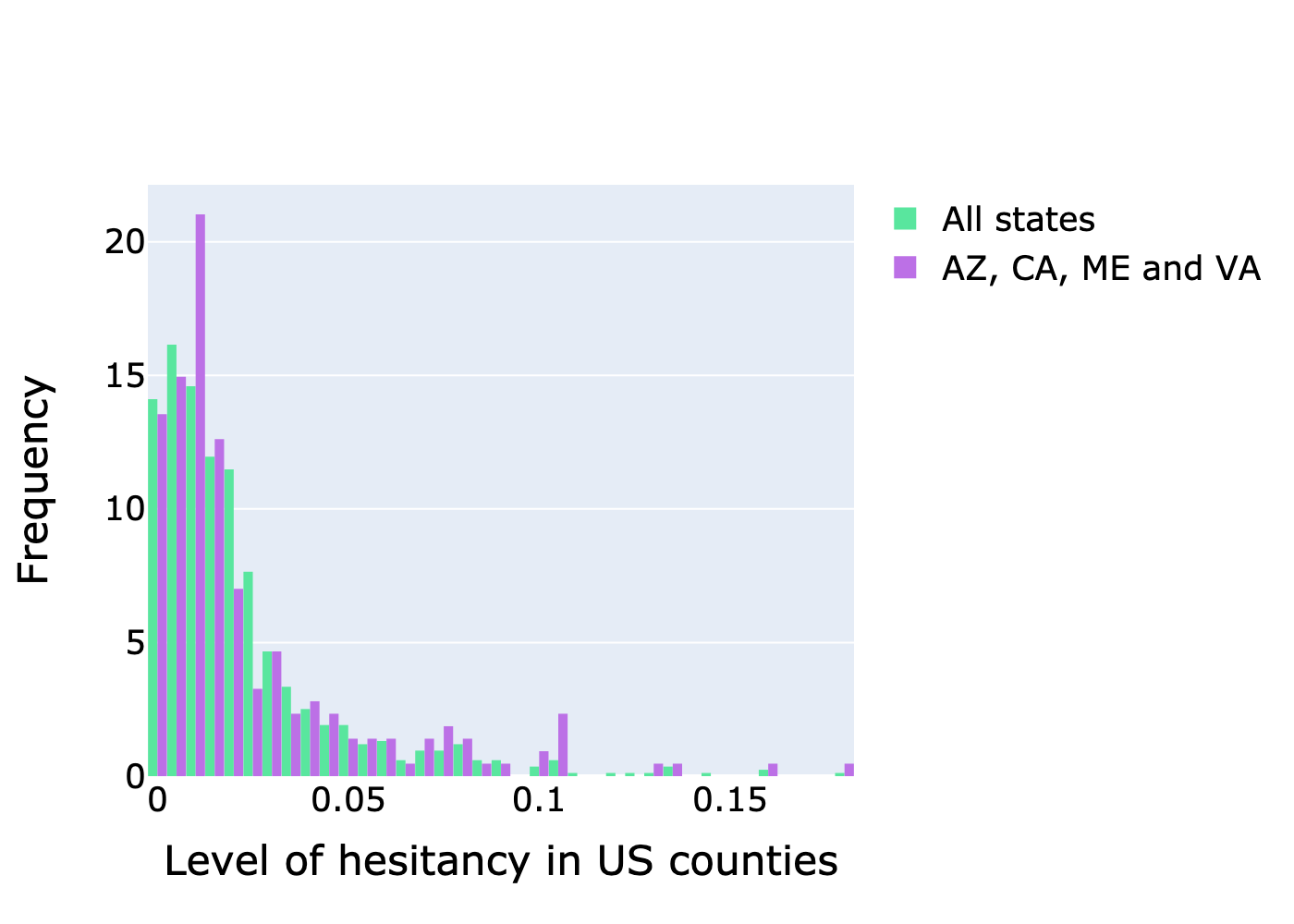

Supplement: S4 Fig — (TIF) [file pcbi.1010437.s004.tif]

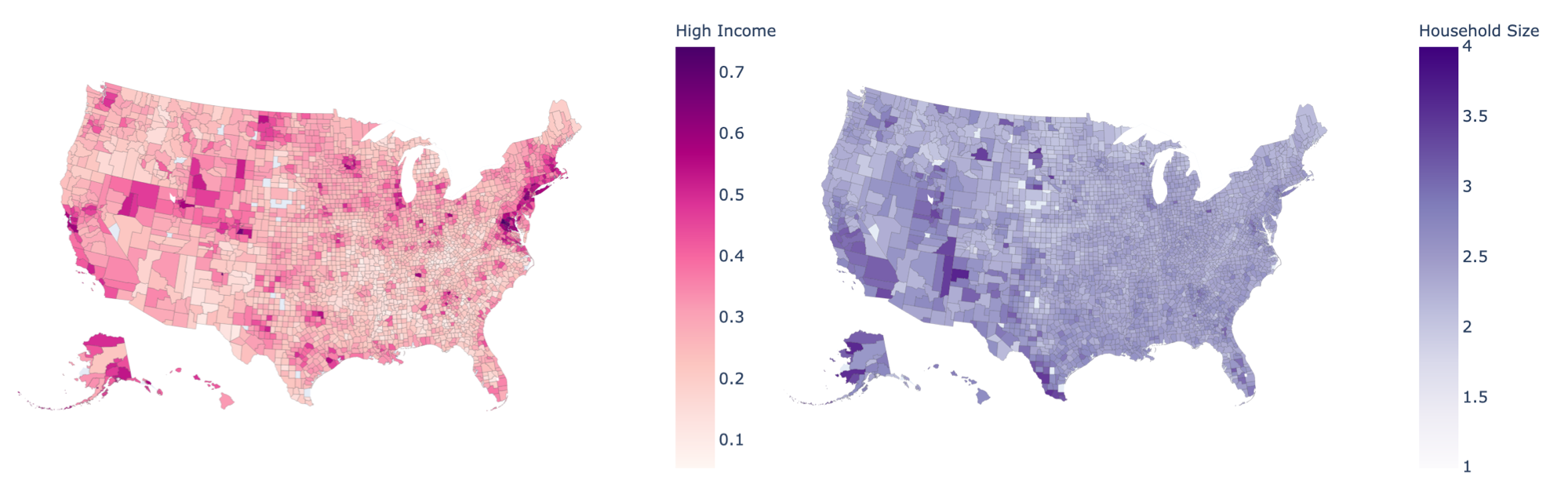

Supplement: S5 Fig — (TIF) [file pcbi.1010437.s005.tif]

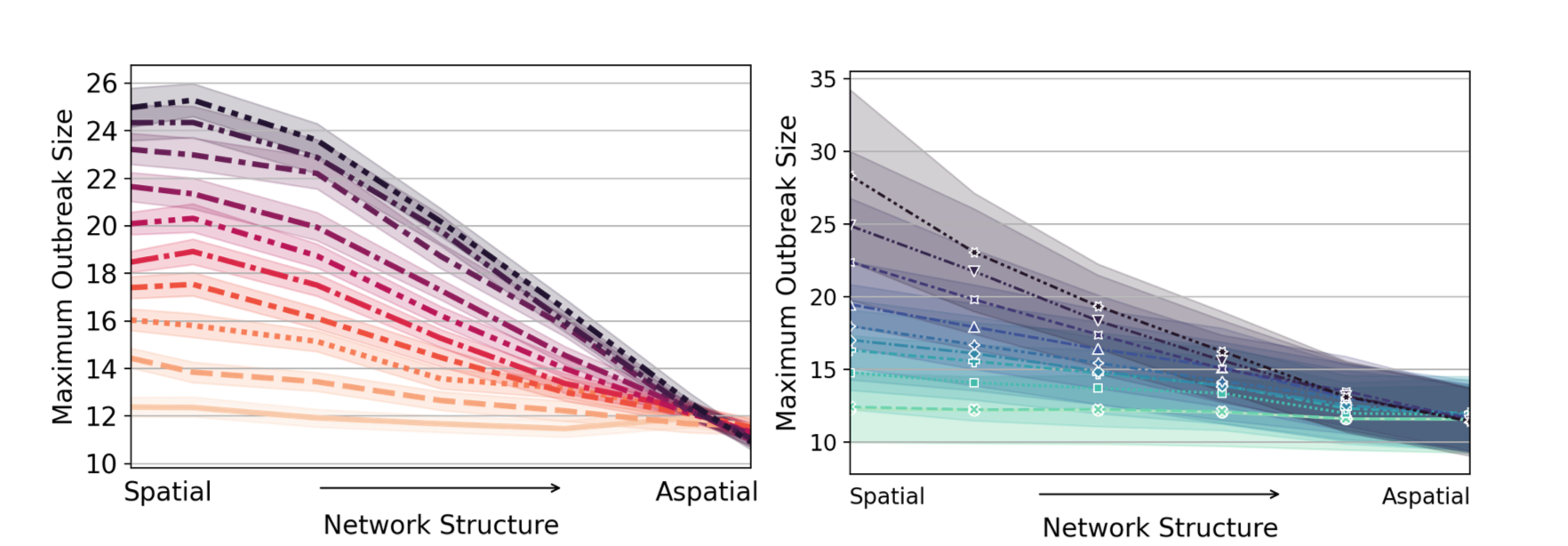

Supplement: S6 Fig — (TIF) [file pcbi.1010437.s006.tif]

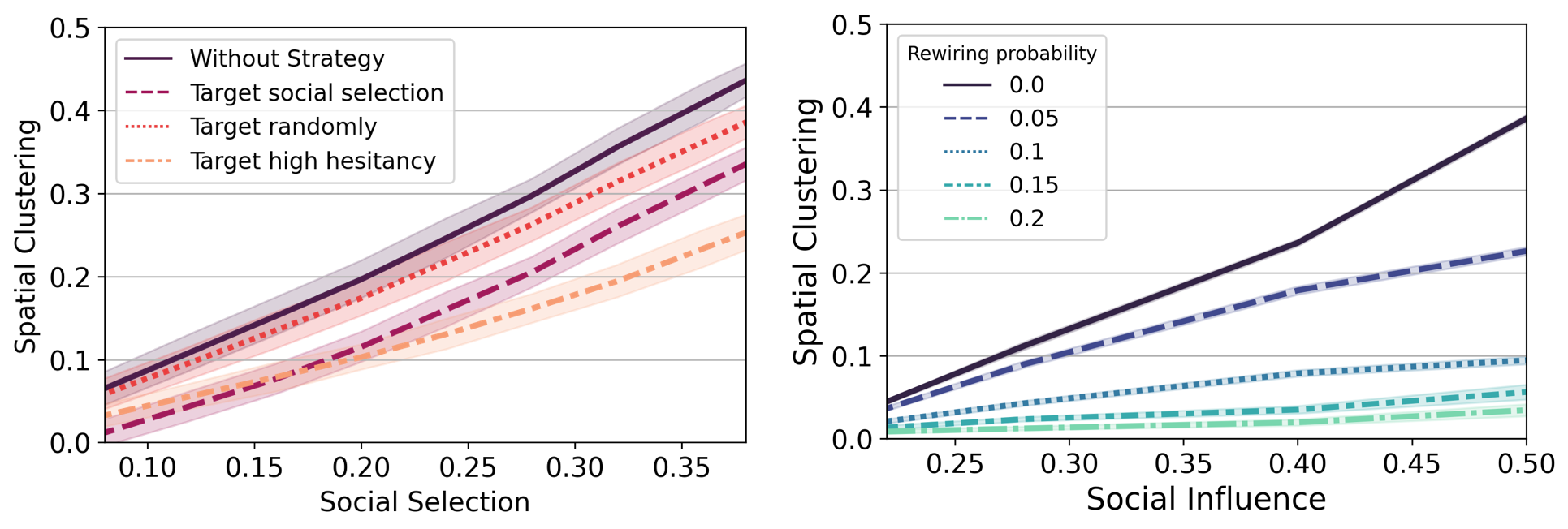

Supplement: S7 Fig — (TIF) [file pcbi.1010437.s007.tif]

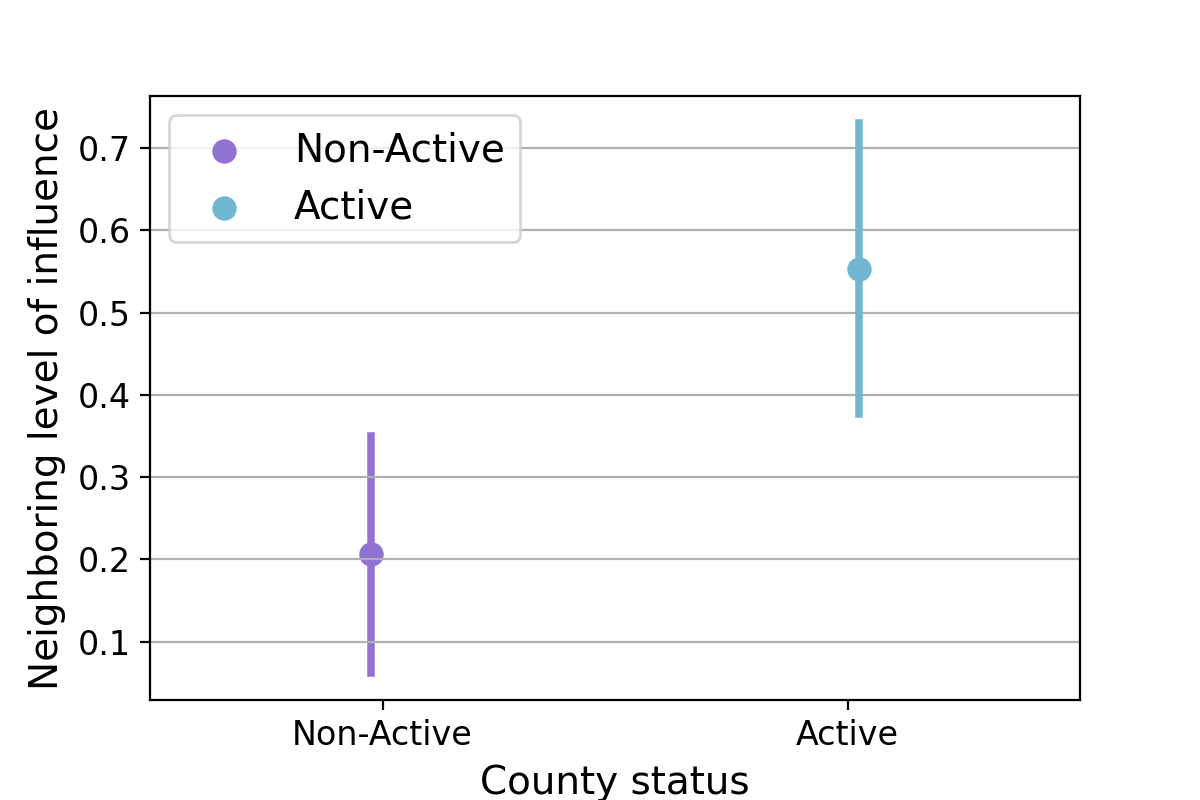

Supplement: S8 Fig — (TIF) [file pcbi.1010437.s008.tif]

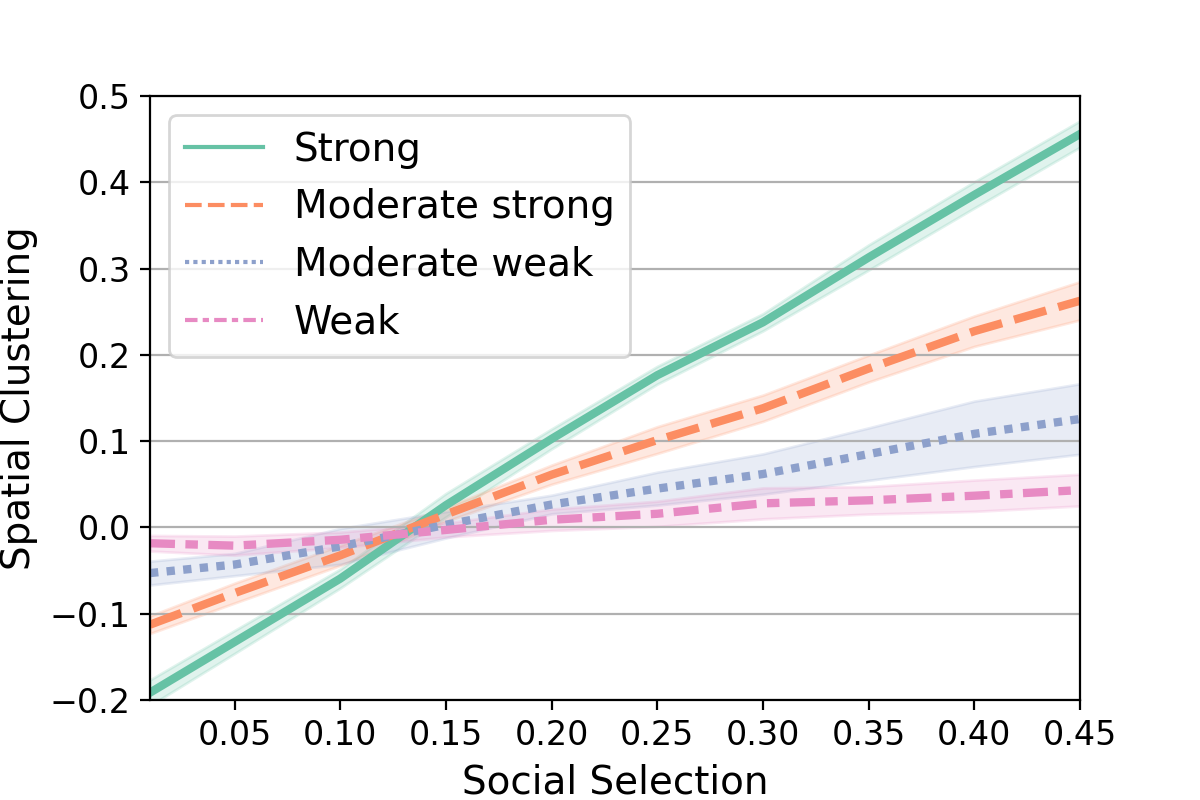

Supplement: S9 Fig — (TIF) [file pcbi.1010437.s009.tif]

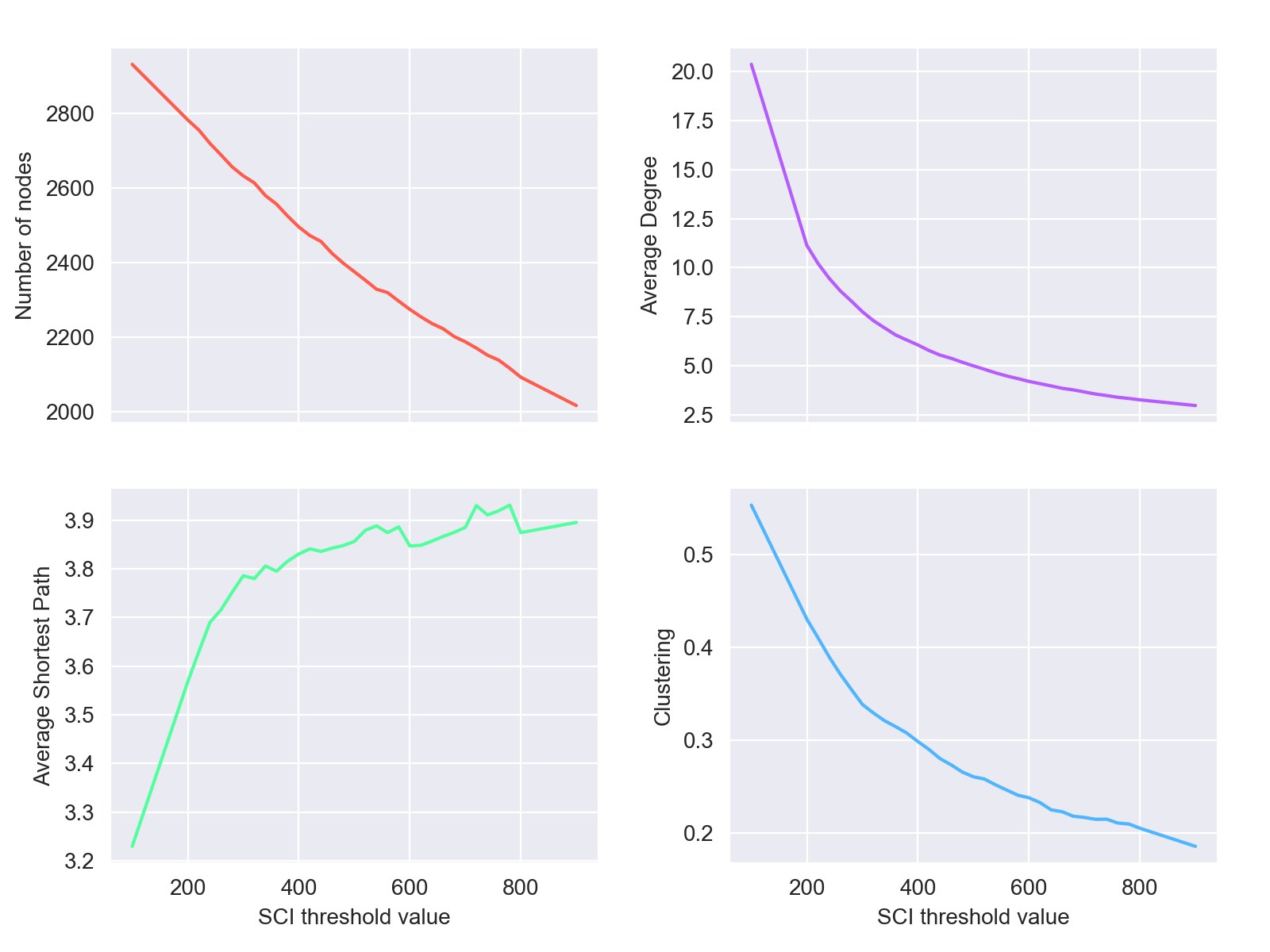

Supplement: S10 Fig — (TIF) [file pcbi.1010437.s010.tif]
