# Supplementary material for: Spatial clustering in vaccination hesitancy: The role of social influence and social selection
Source: PLoS Comput Biol. 2022 Oct 13;18(10):e1010437. doi: 10.1371/journal.pcbi.1010437 (PMC9562150; doi:10.1371/journal.pcbi.1010437)
Supplement: S1 Table — (PDF) [file pcbi.1010437.s011.pdf]

	California	Virginia	Florida	Iowa
Mean	0.0139	0.0082	0.0063	0.0105
Standard Deviation	$\pm$ 0.00141	$\pm$ 0.0023	$\pm$ 0.0043	$\pm$ 0.0014

Table S1: Increment in hesitant behavior for the period of years $2015 - 2018$
